# Comparison of the Efficacy of Q‐Switched Versus Long‐Pulsed Fractional 1064 nm Nd:YAG Laser in the Treatment of Active Facial Acne: A Pilot, Double‐Blind, Randomized, Controlled, Split‐Face Study

**DOI:** 10.1002/hsr2.72354

**Published:** 2026-04-14

**Authors:** Dorian Maghsoodloo, Alireza Jafarzadeh, Mohammadreza Ghassemi, Afsaneh Sadeghzadeh‐Bazargan, Azadeh Goodarzi

**Affiliations:** ^1^ Department of Dermatology, Hazrat Fatemeh Hospital, School of Medicine Iran University of Medical Sciences Asadabadi Street Tehran Tehran Iran

**Keywords:** acne, clinical trial, fractional laser, leeds, Nd:YAG, Q‐switched

## Abstract

**Background and Aims:**

There are different treatment options for acne including topicals, systemic, hormonal, intralesional, and laser treatments. Fractional Nd: YAG 1064 nm laser has been used to treat acne scars and there are also reports on its use in active acne. In this pilot, split‐face, randomized trial conducted as an adjunct to standard care, the aim was to compare effects of long‐pulse (LP) with Quality‐switched (QS) Nd:YAG laser on active acne.

**Methods:**

This is a double‐blind, randomized, controlled, split‐face study comparing LP versus QS Nd: YAG laser for treating active acne in 20 participants (age range, 18–50 years). Patients had 3 sessions of treatment with 3‐week intervals, with a final follow‐up 4 weeks later. Assessments were performed using the modified Leeds revised acne grading system, physician global assessment and patient global satisfaction. All participants received daily oral azithromycin as standardized background therapy; lasers were evaluated as adjunctive treatments.

**Results:**

Both laser modes decreased means of modified Leeds scores at final follow‐up. The percentage change was significantly higher in QS compared to LP group (50% vs. 32%, paired comparison *p* = 0.04). Both physicians and patients were significantly satisfied with the results with both treatments (*p* = 0.001).

**Conclusion:**

As adjuncts to daily azithromycin, both LP and QS 1064 nm Nd:YAG lasers showed reductions in acne severity; QS demonstrated a modest within‐subject advantage. Given the small sample and adjunctive systemic therapy, findings are preliminary and hypothesis‐generating.

## 1 Introduction

Acne vulgaris affects approximately 80% of individuals at some point during life [1], and may lead to post‐inflammatory pigmentation and/or scars and potentially can be psychologically very distressing [2]. Abnormal ductal keratinization, seborrhoea, toll‐like receptor (TLR)2‐induced inflammation, and microbiota all play their roles in acne pathogenesis [3]. There are different treatment options for acne, including topical, oral, and laser/light/energy‐based therapies and they should be tailored to the patient's presentation and needs.

Moderate‐to‐severe acne often needs an amalgamation of topical agents, systemic antibiotics, hormonal agents, or oral isotretinoin [4]. Although lasers and energy‐based treatments do not appear in the American Academy of Dermatology's primary acne treatment guidelines as first‐line monotherapy, they have been used primarily as adjunctive treatments. Modalities such as intense pulsed light (IPL), pulsed dye laser (PDL), and other lasers, such as nonablative fractional lasers, are increasingly recognized as promising interventions for acne [5].

Red‐light and near‐infrared lasers, including the 675 nm wavelength, have been shown to improve inflammatory acne lesions, with evidence suggesting that combination therapy with systemic isotretinoin may accelerate and enhance clinical response compared with laser monotherapy. These findings support the concept of synergistic effects between laser‐based photothermal mechanisms and pharmacologic modulation of sebaceous gland activity [6].

More recently, emerging wavelength‐specific technologies such as the 1726 nm laser have been developed to selectively target sebaceous glands through controlled photothermolysis while minimizing collateral thermal damage [7]. Both experimental and clinical studies have demonstrated that this wavelength can induce durable reductions in inflammatory acne lesions with favorable safety and tolerability profiles across a wide range of skin types, even in moderate‐to‐severe acne. Together, these developments highlight a growing interest in wavelength‐specific and combination laser protocols for acne management and underscore the importance of contextualizing established laser modalities within this evolving therapeutic landscape [8, 9].

Neodimium‐doped yttrium aluminum garnet (Nd:YAG) 1064 nm laser has been used in the treatment of active acne. It has been able to decrease the number of inflammatory lesions, total number of comedones and sebum production compared to control [10]. Quality‐switched (QS) laser can reduce the number of Cutibacterium Acnes, reduce the number of sebaceous glands and help regulate sebum secretion [11].

In this split‐face study we compared the effects of long pulse (LP) 1064 nm Nd:YAG laser with QS 1064 nm Nd:YAG laser on facial active acne.

## 2 Materials and Methods

### 2.1 Study Design and Setting

This study was designed as a split‐face, patient‐ and assessor‐blinded, randomized controlled pilot clinical trial conducted on 20 patients with facial acne at a university hospital in Tehran, Iran. The study followed the principles of the Declaration of Helsinki and was registered at the Iranian Registry of Clinical Trials (IRCT20170809035597N4; registered 10 June 2023).

### 2.2 Ethical Considerations

The study protocol was approved by the Ethics Committee of Iran University of Medical Sciences (IR.IUMS.FMD.REC.1402.074; approved on 13 May 2023). Written informed consent was obtained from all participants prior to enrollment.

### 2.3 Participants

**Figure 1:**
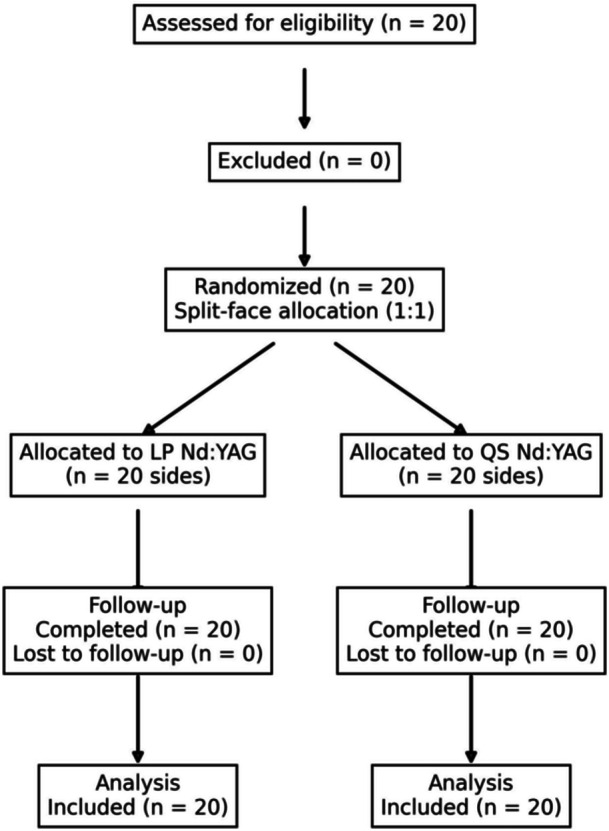
CONSORT flow diagram of the study.

Eligible participants were individuals aged 18–50 years with a dermatologist‐confirmed diagnosis of facial acne vulgaris (Figure 1). Inclusion criteria included no use of systemic acne treatments within the preceding 3 months, absence of uncontrolled systemic disease, and no participation in other clinical trials. Exclusion criteria included hypersensitivity to study interventions, development of viral or bacterial infections during the study, or occurrence of any serious adverse events.

### 2.4 Randomization and Allocation Concealment

A 1:1 left/right facial allocation sequence was generated using a random‐number table by an independent statistician. Allocation was concealed using opaque, sealed envelopes, which were opened at the first treatment session.

### 2.5 Blinding

Due to the nature of the interventions, the treating laser operator could not be blinded. However, participants were blinded to treatment allocation through masking of facial sides and the use of protective eyewear. Outcome assessments were performed by two independent dermatologists who were blinded to allocation.

### 2.6 Interventions

Each participant received both interventions in a split‐face design, with one side treated using long pulsed (LP) Nd:YAG laser and the contralateral side treated using Q‐switched (QS) Nd:YAG laser. The QS laser (Spectra™ XT, MEDI‐INVEST NV, Belgium) operated at a pulse rate of 10 Hz, spot size of 10 mm, and fluence of 1.5 J/cm². The LP laser (Fotona®, Slovenia) operated at a pulse rate of 4 Hz, spot size of 2 mm, fluence of 60 J/cm², and pulse duration of 25 ms. LP treatment endpoints consisted of two passes per lesion, while QS treatment involved delivery of 1200–1500 pulses over the entire treated area.

All participants underwent three treatment sessions at 3‐week intervals, with a final follow‐up 4 weeks after the last session. Digital photographs were obtained at each visit under standardized conditions using a Canon® EOS 750D camera. To standardize background therapy, all participants received oral azithromycin (250 mg daily) throughout the study period. Participants were instructed to avoid other acne treatments, including topical or systemic agents. Standard skincare included daily antibacterial face washing and regular use of sunscreen (SPF ≥ 50). The forehead region was designated as an untreated control area for all participants.

### 2.7 Outcome Measures and Assessment

The primary outcome was the percentage change in modified Leeds acne score from baseline to final follow‐up for each treated side. A modified Leeds revised acne grading system (ordinal scale 1–12) was used to allow more granular assessment, with predefined correspondence to standard acne severity grades.

Both categorical grades and numerical scores were recorded for each region.

Secondary outcomes included:

‐Physician Global Assessment (PGA) using a −2 to +2 scale

(mean of two blinded dermatologists)

‐Patient Global Satisfaction (PGS)

‐Pain assessment using a 10‐point visual analogue scale (VAS)

**‐**Recording of adverse events at each session.

### 2.8 Statistical Analysis

Normality of data distribution was assessed using the Shapiro–Wilk test. The primary analysis compared with in subject differences between QS and LP treatments using paired t‐tests or Wilcoxon signed‐rank tests where appropriate. Repeated‐measures ANOVA (or non‐parametric equivalents) was used to evaluate changes over time. Descriptive statistics were reported as mean (standard deviation) and frequencies.

Given the pilot nature of the study, no adjustment for multiple comparisons was performed, and all p‐values were two‐sided with a significance level of *α* = 0.05. Analyses were conducted using SPSS® version 22 (SPSS Inc., Chicago, IL, USA). No formal sample size calculation was performed; the sample size was based on feasibility and intended for exploratory, hypothesis‐generating analysis.

## 3 Results

Twenty patients (65% females and 35% males) with a mean age of 22.5 ± 3.5 years were selected for this study. All our participants had Fitzpatrick phototypes III (60%) or IV (40%). In each patient, the forehead area was used as control and half‐face received LP Nd:YAG and the other half‐face received QS Nd:YAG. No truncal acne participants were included; truncal control was not used.

### 3.1 Modified Leeds Revised Acne Grading Scores

**Figure 2:**
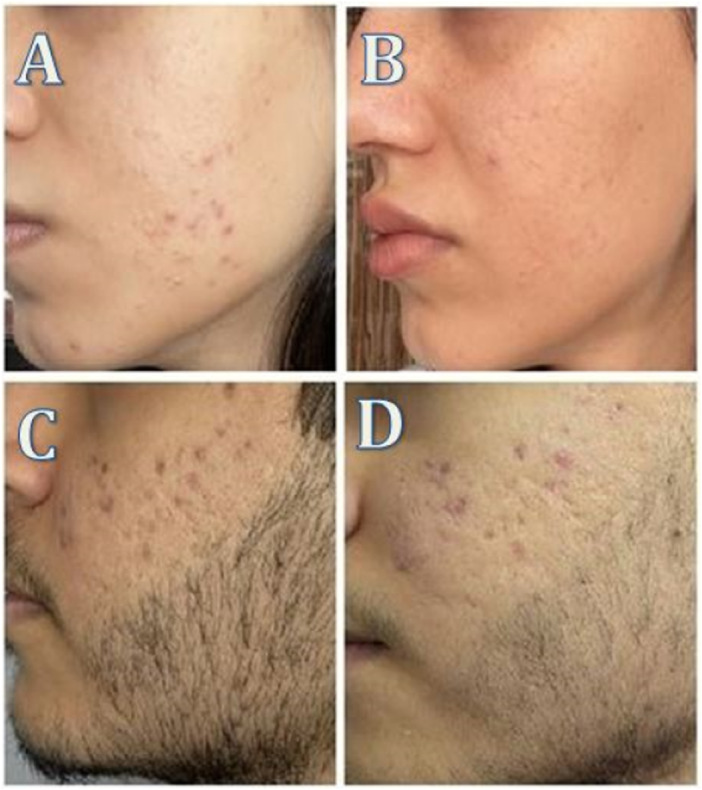
QS Nd:YAG laser results: (A) Before treatment in Patient 1; (B) After treatment in Patient 1; (C) Before treatment in Patient 2; (D) After treatment in Patient 2.

**Figure 3:**
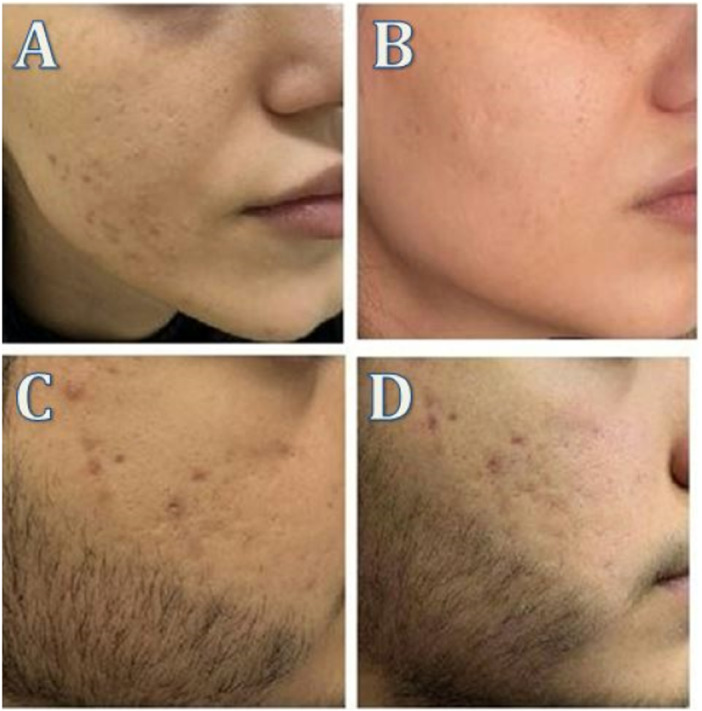
LP Nd:YAG laser results: (A) Before treatment in Patient 1; (B) After treatment in Patient 1; (C) Before treatment in Patient 2; (D) After treatment in Patient 2.

Before study, mean modified Leeds grades in control, LP, and QS groups were 2.25, 3.00, and 2.90, respectively (*p* = 0.11). After first session, they were 2.25, 2.95, and 2.65; differences were not significant. After the second treatment, means were 2.20, 2.57, and 2.00; differences remained non‐significant. At final follow‐up, means were 2.15, 2.05, and 1.45 in control, LP, and QS groups, respectively (*p* = 0.13). We further investigated the percent change in QS vs LP groups; percent change was defined as (baseline minus final)/baseline × 100. At final follow‐up, 50% and 32% change was observed in QS and LP groups, respectively. While both lasers reduced the mean values (Table 1, Figures 2, 3), the reduction was greater with the QS laser. The within‐subject paired comparison of percent change (QS vs. LP) yielded a p‐value of 0.04.

**Table 1:** Modified Leeds Revised Scores in Different Sessions.

Index	Session	Group	Mean	Standard deviation (SD)	95% Confidence intervals		*p*‐value
					Lower band	Upper band	
Modified Leeds revised acne grading system grades	Before intervention	QS	2.90	0.85	2.50	3.30	0.11
		LP	3.00	0.89	2.59	3.41	
		Control	2.25	1.74	1.43	3.07	
		Total	2.72	1.25	2.40	3.04	
	1st	QS	2.65	0.81	2.27	3.03	0.19
		LP	2.95	0.92	2.53	3.37	
		Control	2.25	1.74	1.43	3.07	
		Total	2.62	1.24	2.31	2.94	
	2nd	QS	2.00	0.92	1.57	2.43	0.37
		LP	2.57	0.87	2.18	2.97	
		Control	2.20	1.79	1.36	3.04	
		Total	2.26	1.26	1.94	2.59	
	3rd	QS	1.45	0.51	1.21	1.69	0.13
		LP	2.05	0.74	1.71	2.38	
		Control	2.15	1.84	1.29	3.01	
		Total	1.89	1.20	1.58	2.19	

### 3.2 PGA, PGS, Adverse Events & VAS

At all sessions, PGA mean scores were significantly higher in LP and QS groups compared to control (*p* = 0.001). At the third session, 61.9% (LP) and 65.0% (QS) reported satisfied/very satisfied vs 5.0% in control. PGS showed a similar pattern (*p* = 0.001). At the third session, 42.9% (LP) and 50.0% (QS) were satisfied/very satisfied vs 5.3% control. For LP sides, VAS mean was 1.50, 1.50, and 1.30 (min 1, max 4) for sessions 1–3. For QS sides, VAS mean was 1.50, 1.55, and 1.40 (min 1, max 4). No between‐side VAS differences were detected at any session. All patients tolerated procedures easily. Adverse events were mild/transient. In LP group, post‐inflammatory hyperpigmentation (PIH) occurred in 2 cases at sessions 1–2 and 1 case at session 3. In QS group, prolonged erythema occurred in 2 cases at sessions 1–2 and 1 case at session 3. All resolved within 2 weeks.

## 4 Discussion

Nd:YAG laser has been shown to be effective in treating active acne [11, 12]. In this study, we observed an improvement in acne lesions and a decrease in and modified Leeds revised scores with both LP and QS Nd:YAG lasers. Fractional lasers cause microthermal zones in the skin and stimulate collagen synthesis and tissue regeneration. They are widely used in treating acne scars, skin rhytides and other conditions [13]. A study by Ochi et al. reported that fractional lasers can significantly improve the quality of life in acne patients suffering from acne scars [14].

The main finding was a modest, statistically significant within‐subject advantage in percent Leeds improvement for QS over LP (50% vs 32%; *p* = 0.04). Because all participants received concomitant oral azithromycin, these results should be interpreted strictly as adjunctive effects of laser relative to the contralateral side, not as standalone monotherapies.

Mechanisms of Nd:YAG laser include effects on sebum production through damage to sebaceous glands, normalizing follicular keratinization and corneocyte adhesion, increasing transforming growth factor (TGF)‐ß, decreasing inflammatory cytokines such as interleukin (IL)‐8, mataloproteinase‐9, nuclear factor kß (NFkß), toll‐like receptor (TLR)‐2, and tumour necrosis factor (TNF)‐α [15]. It seems that destructive effect of 1064 nm Nd:YAG laser on dilated superficial vessels in inflammatory acne and changes in secretion of the above‐mentioned cytokines are associated with clinical improvement of inflammatory lesions [16, 17]. The improvement in non‐inflammatory lesions might be due to thermal destruction of sebaceous glands and therefore, decreased sebum production [18]. Our findings are in line with findings from a study by Chalermsuwiwattanakan et al that compared 1064 nm Nd:YAG with 595 nm pulsed‐dye laser (PDL) in treating acne. After 3 treatment session 2 weeks apart, they reported a 50.06% and 15.95% in inflammatory and non‐inflammatory lesions, respectively [11]. Another split‐face study compared LP Nd:YAG with botulinum toxin in treating acne. After 3 monthly LP Nd:YAG sessions, and reported 70.42% and 41.8% improvement in inflammatory and non‐inflammatory lesions, respectively. After 3 months follow up, the improvement rate was increased to 85.72% and 49.05%, respectively [12]. In another study by Monib et al. after 3 sessions of Nd:YAG with 2‐week intervals, reported 65.7% and 44.0% improvement in inflammatory and non‐inflammatory lesions, respectively [16]. In line with our findings, the above‐mentioned studies reported more improvement in inflammatory compared to non‐inflammatory lesions. Mechanisms of fractional lasers include formation of channels from epidermis to dermis (leading to decreased obstruction of sweat glands and transepidermal elimination of sebum), coagulation around the channels (leading to decreased inflammation and vascular structures) [19], residual thermal damage in adjacent tissues (leading to induction of protein denaturation, decreased load of Cutibacterium Acnes, and affecting sebaceous glands) [20, 21], and targeting cystic lesions using its water content as the main chromophore [22]. Cho et al have investigated fractional CO2 laser role in treatment of acne in 7 patients and have reported moderate (26–50%) to significant (> 75%) reduction in acne lesions after 2–3 treatment sessions [23]. Pestoni Porvén et al have also reported 2 cases of improvement in microcytic and nodulocystic acne lesions with only one session of fractional CO2 laser, along with topical retinoid and antibiotics [24]. Shin et al have also reported a decrease in the mean number of papules (54%) and pustules (41%) after 1–2 treatments with fractional CO2 laser [25].

A randomized split‐face study by Hammoda et al. evaluated the comparative therapeutic efficacy of fractional CO₂ and Nd:YAG lasers in 30 patients with acne vulgaris. Each participant received four treatment sessions at 14‐day intervals. Clinical outcomes were assessed through lesion counts and the Global Acne Severity (GEA) Scale. Both treatment modalities resulted in a statistically significant reduction in GEA scores immediately after therapy and at the 3‐month follow‐up. However, the fractional CO₂ laser demonstrated a significantly greater reduction in GEA scores compared to Nd:YAG (*p* = 0.006 and *p* = 0.000, respectively). In addition, fractional CO₂ achieved superior clinical improvement in both inflammatory and noninflammatory lesions (*p* = 0.007 and *p* = 0.000, respectively) and was associated with higher patient satisfaction (*p* = 0.004) [26].

In a prospective randomized split‐face trial, Jing et al. compared the efficacy and safety of a 1064‐nm Nd:YAG picosecond laser equipped with a fractional micro‐lens array (P‐MLA) versus electro‐optical synergy (ELOS) in the treatment of post‐acne erythema (PAE). The study included 20 patients, predominantly with darker skin phototypes (Fitzpatrick III–VI). Each participant received P‐MLA treatment on one facial side and ELOS on the contralateral side, with three sessions performed at 4‐week intervals and follow‐up extending to 12 weeks. Treatment outcomes were evaluated using standardized VISIA imaging, lesion counts, clinical erythema assessment, and patient satisfaction scores, while adverse events were systematically recorded.

Both modalities resulted in significant reductions in erythema. However, the P‐MLA‐treated sides demonstrated a greater decrease in PAE lesion counts, higher clinical response rates at week 12, and more pronounced reductions in VISIA red zone scores compared to ELOS. Additionally, P‐MLA was associated with improvements in skin texture parameters, including wrinkles and pigmentation. Patient satisfaction was high and comparable between groups, with an overall satisfaction rate approaching 95% [27].

Mechanistically, the 1064‐nm Nd:YAG laser penetrates deeply into the dermis and induces selective photothermolysis of hyperactive sebaceous glands while modulating key inflammatory mediators, including TLR‐2, IL‐8, and TNF‐α. This dual action contributes to reduced sebum production and attenuation of inflammatory lesion burden. Its selective dermal targeting, with minimal epidermal disruption, may explain the favorable safety profile and limited downtime associated with Nd:YAG treatment [26, 27].

In contrast, fractional ablative lasers such as fractional CO₂ create controlled microthermal treatment zones that extend from the epidermis into the dermis. These microscopic columns stimulate epidermal renewal, collagen remodeling, and facilitate transepidermal elimination of debris and sebum, resulting in broader dermal restructuring and textural improvement [26, 27].

Regarding adverse events, a study by Monib et al reported transient erythema and oedema which decreased treatment‐to‐about 24 h after treatment [16]. Studies by Rahman et al., Graber et al., and Alster et al. reported that adverse events from fractional Nd: YAG lasers are often mild and transient; the reported adverse events were erythema, dryness, and oedema [28, 29]. In our study, the adverse events included PIH with LP laser and prolonged erythema (all cases resolved after 2 weeks) with QS laser; as in the above‐mentioned studies, these adverse events were mild and transitory. In our study, all cases of PIH occurred in patients with skin types IV; although all cases were mild and transitory, this indicates the need for setting lower fluences and/or higher pulse durations with less number of total passes/pulses for such patients.

We found that QS laser was marginally more effective in reducing acne severity compared to LP laser. This might have resulted from a few factors; first, extremely short bursts of energy in QS laser (nanoseconds vs microseconds in case of LP laser) results in less heat build up in adjacent tissues and minimizes the risk of injury to untreated tissues. Prolonged and excess heat might induce more inflammation and therefore increased possibility of PIH and scars. Cutibacterium Acnes and melanin also absorb these very short but high‐energy pulses in QS lasers and therefore effectiveness increases in targeting one of the pathophysiologic agents in acne formation and reducing the risk of PIH, which usually accompanies inflamed lesions [30, 31].

## 5 Limitations

This study has several important limitations that should be considered when interpreting the findings. First, this was a pilot study with a relatively small sample size and no a priori power calculation; therefore, the statistical power is limited and the results should be interpreted as exploratory and hypothesis‐generating rather than definitive. The statistically significant difference observed between Q‐switched and long‐pulsed Nd:YAG lasers may represent a false‐positive finding and requires confirmation in larger, adequately powered trials.

Second, all participants received concomitant systemic antibiotic therapy (oral azithromycin) as standardized background treatment. Although this approach was intended to symmetrically control systemic inflammatory influences across both facial sides, it represents a major confounding factor and prevents isolation of the independent therapeutic effects of each laser modality. Accordingly, the lasers should be interpreted strictly as adjunctive treatments, not as standalone therapies.

## 6 Conclusion

This study suggests that both LP and QS 1064 nm Nd:YAG lasers used adjunctively with standardized systemic azithromycin may reduce acne severity compared to untreated control areas, with a modest within‐subject advantage of QS over LP in percent improvement. Given pilot design, small sample, and systemic confounding, conclusions are preliminary; larger, adequately powered trials—ideally without concurrent systemic antibiotics—are needed to confirm these signals and define clinical impact.

## 7 Declarations

All authors have read and approved the final version of the manuscript. Dr. Azadeh Goodarzi had full access to all of the data in this study and takes complete responsibility for the integrity of the data and the accuracy of the data analysis.

## Author Contributions

**Dorian Maghsoodloo:** investigation, validation, methodology. **Alireza Jafarzadeh:** writing – review and editing, project administration. **Mohammadreza Ghassemi:** visualization, validation; data curation. **Afsaneh Sadeghzadeh‐Bazargan:** supervision, resources. **Azadeh Goodarzi:** project administration, conceptualization, writing – review and editing.

## Funding

The authors have nothing to report.

## Ethics Statement

The researchers were committed to and adhered to the principles of the Helsinki Convention and the Ethics Committee of the Iran University of Medical Sciences (IR. IUMS. FMD. REC.1402.074) throughout all stages of the study. The study was also registered with the Iranian Registry of Clinical Trials (IRCT20170809035597N4; registered 10 June 2023).

## Conflicts of Interest

The authors declare no conflicts of interest.

## Transparency Statement

The lead author Azadeh Goodarzi affirms that this manuscript is an honest, accurate, and transparent account of the study being reported; that no important aspects of the study have been omitted; and that any discrepancies from the study as planned (and, if relevant, registered) have been explained.

## Data Availability

The data that support the findings of this study are available from the corresponding author upon reasonable request.
